# Psychological mechanisms of healthy lifestyle and academic burnout: a moderated mediation model

**DOI:** 10.3389/fpsyg.2025.1533693

**Published:** 2025-03-21

**Authors:** Jiantao Lu, Yu Wang, Xinjing Liu, Qian Zhang, Yuqin Yan

**Affiliations:** ^1^School Hospital, Qilu University of Technology (Shandong Academy of Sciences), Ji’nan, Shandong, China; ^2^School of Psychology, Shandong Normal University, Ji’nan, Shandong, China; ^3^School Hospital, Shandong University of Science and Technology, Jinan, Shandong, China

**Keywords:** healthy lifestyle, academic burnout, dormitory conflict coping style, gender, college student

## Abstract

**Objective:**

Academic burnout is a prevalent phenomenon among college students. According to the Conservation of Resources Theory, when there is an imbalance between invested resources and expected returns, individuals may suffer from academic or job burnout. If an individual has sufficient resources, these resources may relieve the negative problem. Healthy lifestyle is believed to improve brain health and provide resources. However, limited research has examined the psychological mechanisms that link academic burnout and a multidimensional healthy lifestyle.

**Methods:**

A sample of 1,186 undergraduate students from freshmen to seniors were recruited using the stratified cluster random sampling method. The participants completed online questionnaires that assessed the healthiness of their lifestyle, dormitory conflict coping style, and academic burnout in April 2021. Bivariate correlation and a moderated mediation model were constructed to examine the relationships among these variables.

**Results:**

The results indicated that (1) healthy lifestyle was negatively correlated with academic burnout (*r* = −0.496, *p* < 0.001), coping styles of competition (*r* = −0.281, *p* < 0.001) and avoidance (*r* = −0.210, *p* < 0.001), but positively correlated with coping styles of cooperation (*r* = 0.342, *p* < 0.001) and obedience (*r* = 0.134, *p* < 0.001); (2) academic burnout was positively correlated with coping styles of competition (*r* = 0.331, *p* < 0.001) and avoidance (*r* = 0.305, *p* < 0.001) and negatively correlated with coping styles of cooperation (*r* = −0.227, *p* < 0.001); and (3) the direct path of healthy lifestyle on academic burnout was partially mediated by coping styles of competition (effect = −0.04, 95%CI: [−0.05,-0.03]) and avoidance (effect = −0.03, 95%CI: [−0.04,-0.02]), which was moderated by gender (*β* = −0.48, *p* = 0.007).

**Conclusion:**

The findings offer valuable insights into the psychological mechanisms underlying the association between academic burnout and multidimensional healthy lifestyle among general college students, as indicated that college students with healthier lifestyles tend to use fewer coping styles of competition and avoidance, leading to a lower level of academic burnout. Such effect is more pronounced among female college students. This study provides a new perspective for the prevention and intervention in college students’ academic burnout.

## 1 Introduction

Academic burnout, which involves the negative attitude and behavior caused by learning pressure or a lack of interest in learning, is common among college students (Lian et al., 2005). Academic burnout reflects a negative psychological attitude toward learning, with 38.1–59.9% of college students experiencing varying degrees of academic burnout (Li et al., 2021; Liu et al., 2023). The detection rate for academic burnout varies by college major, especially among nursing students, who have a high rate of academic burnout (Liu et al., 2023). Academic burnout can affect students’ academic performance as well as their mental health by reducing life satisfaction and academic efficacy and eliciting anxiety and depression in addition to various adverse behavioral reactions (Bask and Salmela-Aro, 2013; Obekpa et al., 2020; Wang et al., 2015; Wang et al., 2022). College students in the transition from late adolescence to early adulthood face competitive academic and employment environment and thus are prone to academic burnout. Therefore, understanding the psychological mechanisms underlying college students’ academic burnout is important for improving their mental health and promoting sustainable development.

According to the Conservation of Resources Theory, when there is an imbalance between invested resources (e.g., psychological resources, social resources and physical energy) and expected returns, individuals will suffer from resource exhaustion and then may arise academic or job burnout (Hobfoll, 1989). Previous studies have revealed a significant negative correlation between learning engagement and academic burnout (Cong et al., 2024; Yan, 2021). Furthermore, students may reduce their invested resources when they perceive that the effort invested is not proportional to the expected returns from learning (Liu et al., 2023). If an individual has insufficient resources, academic or job burnout may arise. A healthy lifestyle is important for improving psychological resources and physical energy, which are negatively correlated with psychological stress (Ezoe and Morimoto, 1994), work stress (Lee et al., 2011), and academic stress (Zhu et al., 2024). A healthy lifestyle, which includes healthy diet, exercise, stress management and so on, is also considered to improve brain health and reduce depression and stress (Cheung and Yip, 2016; Moriarty et al., 2021). Interventions that improve awareness of health could relieve young adults’ negative mental state (Tsai et al., 2021), while a positive mental state could reduce the level of academic burnout (Tang et al., 2021; Yan, 2021). A meta-analysis revealed that academic burnout can be relieved by interventions that include a healthy lifestyle based on physical activity, health coaching, and relaxing activities (Puente-Hidalgo et al., 2024). Physical exercise and academic burnout were negatively correlated (Chen et al., 2022; Fu et al., 2023; Jin et al., 2024; Rehman et al., 2024). There is also evidence that academic burnout among undergraduate nursing students is negatively correlated with healthy lifestyles (Naderi et al., 2021). However, considering that a healthy lifestyle includes not only physical activity or exercise but also a healthy diet, regular living, stress management, and so on, there is still a lack of data on the relationship between an overall healthy lifestyle and academic burnout. Therefore, it is essential to examine the relationship between academic burnout and a multidimensional healthy lifestyle among general college students.

The underlying mechanisms connecting a healthy lifestyle and academic burnout remain unclear. Transactional Theory of Stress proposes that coping style, which refers to the emotional and behavioral responses to stress, is a mediating factor between physical and mental health and stressful situation; it regulates the understanding of life events and relieves stress (Lazarus and Folkman, 1987). Cooperation, obedience, avoidance and competition are common coping styles for conflict among college students (Coiro et al., 2017; Deng et al., 2015). Cooperation is a positive coping style, while avoidance and competition are negative coping styles (Chen et al., 2024; Deng et al., 2015; Yang et al., 2021). Empirical evidence suggests that positive coping styles (e.g., cooperation coping style) could relieve academic burnout (Chae and Lee, 2017), whereas negative coping styles (e.g., competition and avoidance coping styles) are likely to lead to academic burnout (Chae and Lee, 2017; Hou, 2023; Puspaningrum and Ruby, 2024; Shi et al., 2023). Moreover, positive coping styles could affect individuals’ mental health and could contribute to healthy behaviors (Zou et al., 2017). Improving stroke patients’ cognition could promote their use of positive coping styles for stress, thereby facilitating healthy behaviors (Liu et al., 2021). Studies of healthy individuals have also shown that positive coping styles could promote healthy lifestyles (Fan et al., 2023). Physical exercise is positively associated with positive coping styles and negatively associated with negative coping styles (e.g., avoidance coping style), which leads to a lower level of psychological distress (Tada, 2017). Cooperation could provide opportunities for mutual support and group encouragement, whereas competition could impair of healthy behaviors (Orji et al., 2019). Therefore, if individuals’ lifestyles are unhealthy, they are likely to perceive greater subjective stress and psychological pressure, which may prompt them to adopt negative coping styles. Moreover, Chinese college students spend most of their spare time in dormitories, and their coping styles for dormitory conflicts may be influence academic burnout and a healthy lifestyle. Previous theories and research have preliminarily revealed that coping styles for dormitory conflict may be a mediating factor between healthy lifestyle and academic burnout, however, no studies have examined this relationship. Therefore, the current study explored how college students’ healthy lifestyle affected academic burnout through dormitory conflict coping styles.

Gender differences in peer groups result in different modes of social interaction that have different adaptive consequences (Liu N. et al., 2024; Liu X. et al., 2024; Xie et al., 2023). Gender differences in coping styles have been found to be inconsistent. Men are more inclined to adopt a competition coping style than women are (Zhao et al., 2019). However, another study found that men are more inclined toward cooperation and obedience than women are, although no significant difference are observed in competition and avoidance between men and women (Asma, 2024). It should be noted that gender is an important factor in healthy lifestyles and academic burnout. Women exhibit healthier lifestyle than men do, while men have higher levels of academic burnout than women do (He et al., 2023; Liu et al., 2023; Wang et al., 2009). Specifically, men demonstrate unhealthier lifestyles than women, which may lead to a higher level of academic burnout. The current study examined whether gender moderated the relationship between healthy lifestyle and academic burnout.

**Figure 1:**
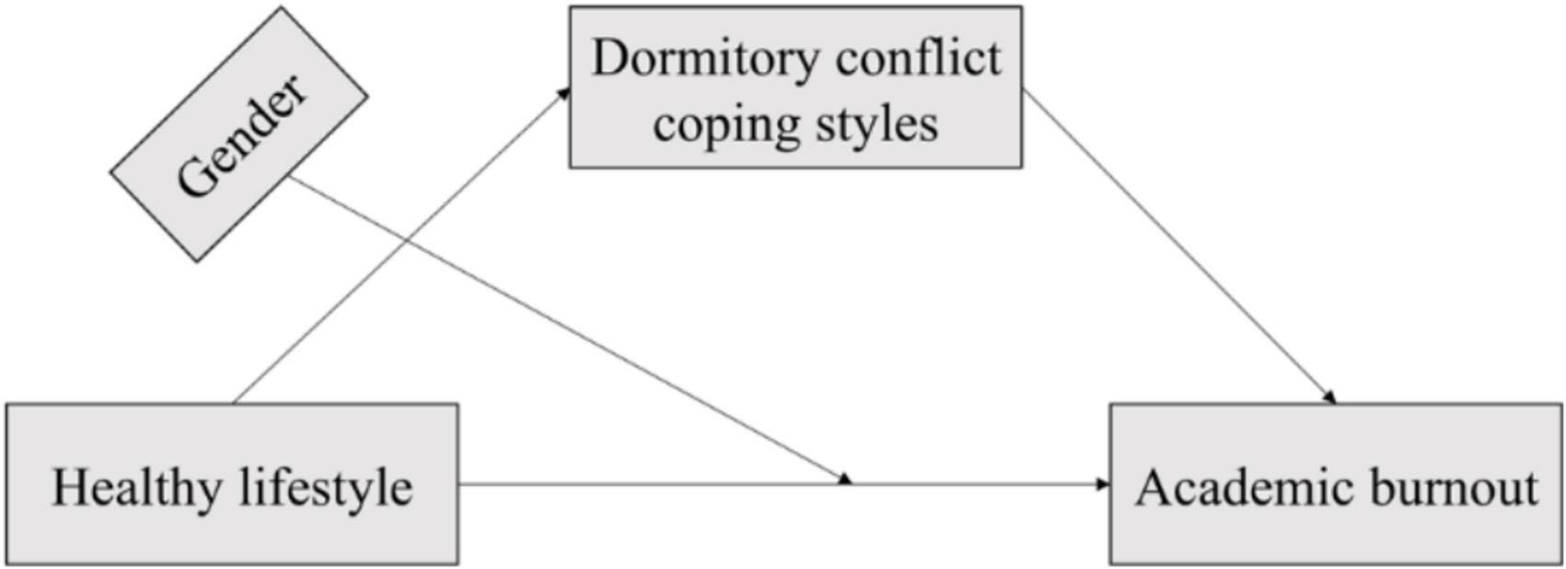
Research framework.

To address the above issues, a moderated mediation model was constructed based on Conservation of Resources Theory and Transactional Theory of Stress (Figure 1). Three hypotheses were proposed. Hypothesis 1: college students with healthier lifestyle exhibit lower level of academic burnout. Hypothesis 2: healthy lifestyle influences academic burnout through the mediating role of dormitory conflict coping styles. Hypothesis 3: gender has a moderating effect on the relationship between healthy lifestyle and academic burnout.

## 2 Methods

### 2.1 Participants and procedure

Using the stratified cluster random sampling method, participants were recruited from the university community through online surveys in April 2021. A total of 1,232 college students completed the survey questionnaire. The questionnaires took approximately 9 min to finish. Inclusion criteria required participants to be undergraduate students from freshman to senior year. Exclusion criteria included: (1) the same option selected consecutively across all questionnaires; (2) outliers assessed using Z-scores (Tabachnick and Fidell, 2007); (3) the completion time less than 1.5 min according to our pilot test to complete the questionnaire. The final valid sample size was 1,186 (456 males, age range: 18–23 years), including 791 freshmen, 284 sophomores, 75 juniors, and 36 seniors. The present study obtained approval from the ethics committee of Qilu University of Technology.

The sample size was based on the previous comparable studies and a power analysis. Previous studies that investigated the relationship between physical exercise and academic burnout in college students have reported sample sizes ranging from 596 to 1,270 participants (Chen et al., 2022; Rehman et al., 2024). Additionally, a power analysis conducted using G*Power 3.1 (Faul et al., 2009) indicated that a sample size of 98 participants would be required to detect a medium effect size f ^2^ of 0.15 (Cohen, 1988) with 80% power at the 0.05 significance level.

### 2.2 Measures

#### 2.2.1 Academic burnout scale

The current study employed the College Students’ Academic Burnout Scale developed by Lian et al. (2005) to evaluate the level of academic burnout among college students. The scale consisted of 20 items, of which 8 items (1, 3, 6, 8, 11, 13, 15, 18) were scored in reverse. Participants were asked to rate each item from “completely disagree” to “completely agree” using a five-point scale. The score of all items was calculated, with higher scores indicating higher levels of academic burnout. The measurement results showed good internal consistency in the current study (Cronbach’s *α* = 0.873).

#### 2.2.2 Healthy lifestyle assessment scale

The healthiness of college students’ lifestyles was measured using the Healthy Lifestyle Assessment Scale developed by Jiao and Wang (2013). This scale included 33 items and measured eight behaviors that were physical exercise, healthy diet, health responsibility, regular life, health hazards, interpersonal behavior, stress management and life appreciation. Participants were asked to rate each item on a scale of “never, occasionally, about half the time, frequently, or always” on a five-point scale. The score of all items was calculated, with higher scores indicating healthier lifestyles. The measurement results showed good internal consistency in the current study (Cronbach’s *α* = 0.907).

#### 2.2.3 Dormitory conflict coping style questionnaire

The current study employed the Dormitory Conflict Coping Style Questionnaire developed by Jia (2009) to evaluate college students’ coping styles for dormitory conflict. The questionnaire included 17 items in four dimensions: competition, cooperation, avoidance, and obedience. The participants were asked to rate each item from “never use” to “often use” on a four-point scale. Higher scores indicated more frequent use of that coping style for conflict. The Cronbach’s α coefficients of each dimension in the current study were 0.757, 0.870, 0. 626, and 0. 606, which is in line with the values of Jia (2009).

### 2.3 Data analysis

The descriptive statistical analysis and correlation analysis of the main variables were conducted with SPSS 21.0. Gender differences among the main variables were analyzed using independent samples *t* tests. One-way ANOVA was used to analyze the effects of grade and place of resident among the main variables, and bivariate correlation analyses were used to analyze the interrelationships among the main variables. The PROCESS 3.0 macro developed by Hayes (2013) was used to estimate the moderated mediation model by using Model 5. The bootstrapping procedure (with 5,000 bootstrap samples) was used to evaluate the 95% confidence interval (CI). It is considered to have a significant indirect effect when the 95% CI does not include zero. The amount of mediating effect was calculated by the indirect effect as a proportion of the total effect.

Harman’s single-factor analysis revealed that the maximum factor explaining the variance was 19.13%, which was below the threshold of 40% (Podsakoff et al., 2003). Therefore, there was no significant common method bias.

## 3 Results

### 3.1 General descriptive statistics and analysis of variance

Descriptive statistics are shown in Table 1. Females’ lifestyle was healthier than those of males, and their academic burnout was lower than those of males. Males used coping styles of competition and avoidance more frequently than females did, while they used coping style of cooperation less frequently than females did. There were no significant differences in grade and place of resident among the main variables.

**Table 1:** Differences in demographic variables for healthy lifestyle, each dormitory conflict coping style, and academic burnout [*M*(*SD*)].

		Healthy lifestyle	Competition	Cooperation	Avoidance	Obedience	Academic burnout
Gender	Male (*n* = 456)	116.15 (18.78)	6.50 (2.33)	13.19 (3.68)	7.83 (2.37)	9.36 (2.40)	55.07 (10.87)
	Female (*n* = 730)	118.66 (15.25)	5.34 (1.70)	13.74 (3.53)	7.48 (2.26)	9.18 (2.05)	52.46 (10.65)
	*t* value	−2.52*	9.93***	−2.56*	2.54*	1.38	4.07***
Grade	Freshman (*n* = 791)	118.24 (16.81)	5.83 (2.07)	13.57 (3.52)	7.65 (2.35)	9.25 (2.23)	53.54 (11.14)
	Sophomore (*n* = 284)	117.22 (17.03)	5.75 (2.01)	13.66 (3.85)	7.49 (2.29)	9.37 (2.25)	53.66 (10.33)
	Junior (*n* = 75)	115.40 (16.43)	5.59 (1.68)	12.80 (3.48)	7.83 (2.16)	8.99 (1.67)	52.01 (9.12)
	Senior (*n* = 36)	114.39 (12.42)	5.53 (1.68)	13.17 (3.52)	7.28 (1.85)	8.75 (1.71)	53.22 (10.43)
	*F* value	1.29	0.58	1.31	0.83	1.29	0.50
Place of resident	Urban (*n* = 397)	118.38 (17.26)	5.83 (2.10)	13.44 (3.80)	7.65 (2.34)	9.29 (2.35)	53.14 (11.90)
	Suburban (*n* = 241)	117.42 (16.20)	5.86 (2.02)	13.37 (3.65)	7.63 (2.40)	9.29 (2.25)	54.19 (9.85)
	Rural (*n* = 548)	117.33 (16.58)	5.72 (2.02)	13.66 (3.42)	7.58 (2.24)	9.20 (2.05)	53.37 (10.37)
	*F* value	0.42	0.50	0.71	0.12	0.28	0.74

*p < 0.05; ***p < 0.001.

### 3.2 Correlation analysis

The results of the correlation analysis are shown in Table 2. Healthy lifestyle was negatively correlated with academic burnout, coping styles of competition and avoidance, while positively correlated with coping styles of cooperation and obedience. Coping styles of competition and avoidance were positively correlated with academic burnout, whereas coping style of cooperation was negatively correlated with academic burnout.

**Table 2:** Correlation analysis of healthy lifestyle, academic burnout, and dormitory conflict coping styles.

	1	2	3	4	5	6
1. Healthy lifestyle	—					
2. Competition	−0.281^***^	—				
3. Cooperation	0.342^***^	−0.165^**^	—			
4. Avoidance	−0.210^***^	0.536^***^	−0.154^***^	—		
5. Obedience	0.134^***^	0.132^***^	0.464^***^	0.186^***^	—	
6. Academic burnout	−0.496^***^	0.331^***^	−0.227^***^	0.305^***^	−0.0002	—

**p < 0.01, ***p < 0.001.

### 3.3 Test of the moderated mediation model

To further investigate whether the relationship between healthy lifestyle and academic burnout is affected by dormitory conflict coping styles and gender, we employed Model 5 from PROCESS for SPSS to analyze the moderated mediating effect. All variables were standardized and three regression equations were estimated. Equation 1 was used to examine whether the direct effect of healthy lifestyle on academic burnout was moderated by gender (Table 3). Equation 2 was used to examine the effect of healthy lifestyle on each dormitory conflict coping style (Table 4). Equation 3 was used to examine the mediating effect of each dormitory conflict coping style on the relationship between healthy lifestyle and academic burnout (Table 3).

**Table 3:** Moderated mediation model test of healthy lifestyle and academic burnout (Equations 1, 3).

	Equation 1				Equation 3			
	*β*	*t*	*p*	95%CI	*β*	*t*	*p*	95%CI
Healthy lifestyle	−0.42	−11.64	<0.001	[−0.32, −0.23]	−0.35	−9.50	<0.001	[−0.27, −0.18]
Gender	0.39	2.22	0.026	[1.00, 16.12]	0.45	2.65	0.008	[2.56, 17.22]
Healthy lifestyle × Gender	−0.48	−2.72	0.007	[−0.15, −0.03]	−0.50	−2.91	0.004	[−0.15, −0.03]
Competition					0.12	3.82	<0.001	[0.30, 0.94]
Avoidance					0.14	4.62	<0.001	[0.37, 0.91]
Obedience					0.04	1.37	0.170	[−0.08, 0.47]
Cooperation					−0.06	−1.86	0.064	[−0.34, 0.01]
*R* ^2^	0.26				0.31			
*F* (*df*)	136.40 (1185)				75.43 (1185)			

**Table 4:** Moderated mediation model test of healthy lifestyle and academic burnout (Equation 2).

	Competition				Avoidance				Obedience				Cooperation			
	*β*	*t*	*p*	95%CI	*β*	*t*	*p*	95%CI	*β*	*t*	*p*	95%CI	*β*	*t*	*p*	95%CI
Healthy lifestyle	−0.29	−7.38	<0.001	[−0.04, −0.03]	−0.23	−5.55	0.001	[−0.04, −0.02]	0.13	3.05	0.002	[0.01, 0.03]	0.28	7.10	<0.001	[0.04, 0.08]
Gender	−0.41	−2.19	0.029	[−3.25, −0.18]	−0.20	−0.99	0.321	[−2.75, 0.80]	−0.13	−0.63	0.530	[−2.33, 1.20]	−0.35	−1.87	0.062	[−5.34, 0.13]
Healthy lifestyle* Gender	0.16	0.81	0.416	[−0.01, 0.02]	0.14	0.70	0.482	[−0.01, 0.02]	0.08	0.38	0.704	[−0.01, 0.02]	0.42	2.16	0.031	[0.002, 0.05]
*R* ^2^	0.15				0.05				0.02				0.12			
*F* (*df*)	67.31 (1185)				19.87 (1185)				8.25 (1185)				55.25 (1185)			

**Figure 2:**
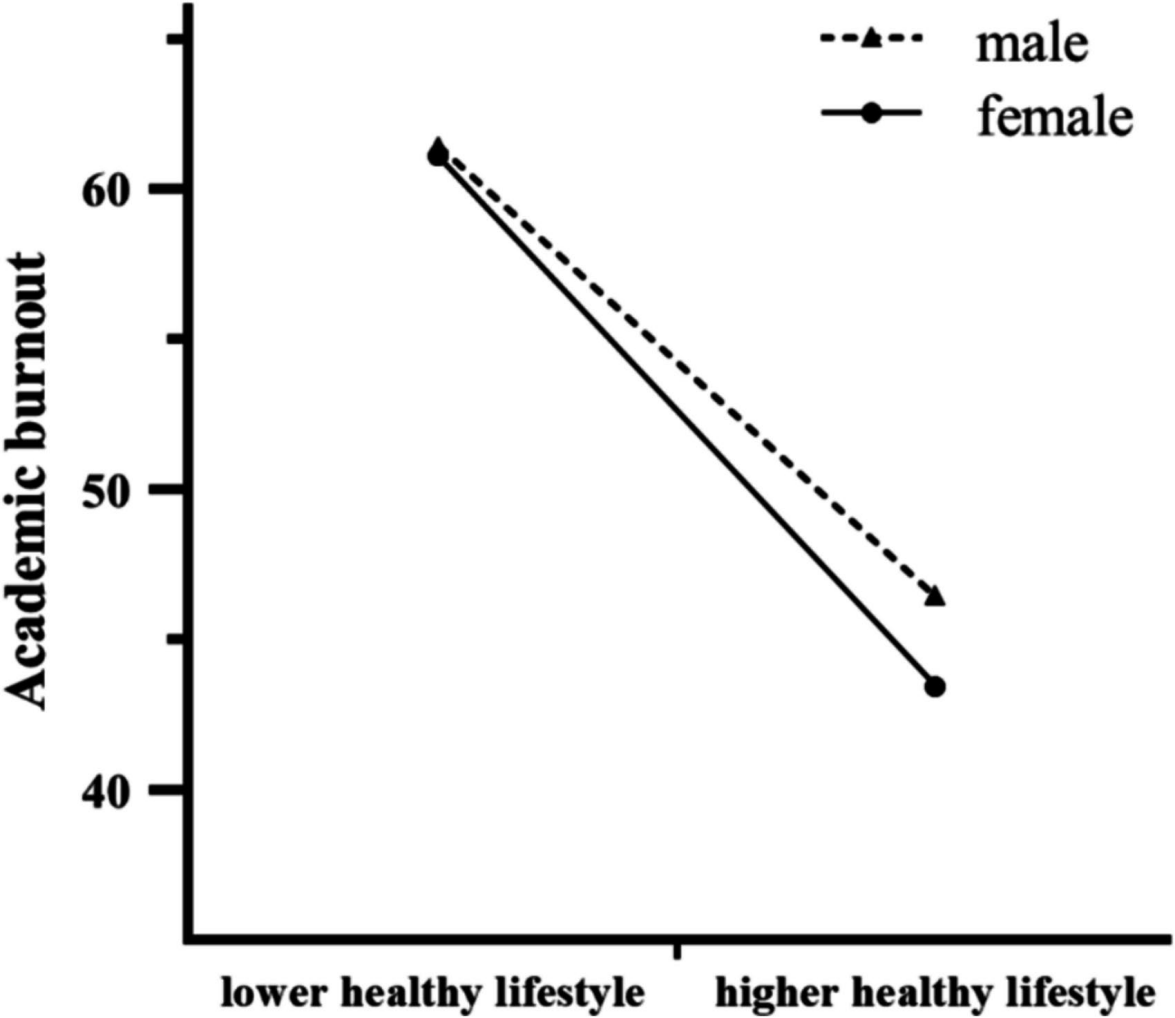
Interaction effect of healthy lifestyle and gender on academic burnout. High and low levels of healthy lifestyle represent one standard deviation above and below the mean.

First, the regression results revealed that, according to Equation 1, healthy lifestyle was negatively associated with academic burnout in college students (*β* = −0.42, *p* < 0.001). Both gender and its interaction with healthy lifestyle also had a significant negative effect on academic burnout (β_1_ = 0.39, *p*_1_ = 0.026; β_2_ = −0.48, *p*_2_ = 0.007). Figure 2 illustrates that the slope of the association between healthy lifestyle and academic burnout was relatively weak for males (β_male_ = −0.47, *t* = −11.29, *p* < 0.001), whereas the slope was relatively strong for females (β_female_ = −0.52, *t* = −16.19, *p* < 0.001). Second, according to Equation 2, healthy lifestyle was positively related to coping styles of obedience (*β* = 0.13, *p* = 0.002) and cooperation (*β* = 0.28, *p* < 0.001), while negatively related to coping styles of competition (*β* = −0.29, *p* < 0.001) and avoidance (*β* = −0.23, *p* = 0.001). Gender was negatively related to coping style of competition (*β* = −0.41, *p* = 0.029), and its interaction with healthy lifestyle had a significant positive effect only on coping style of cooperation (*β* = 0.42, *p* = 0.031). Third, according to Equation 3, healthy lifestyle was negatively associated with academic burnout of college students (*β* = −0.35, *p* < 0.001). The interaction between gender and healthy lifestyle was negatively associated with academic burnout (*β* = 0.45, *p* = 0.008). Both coping styles of competition and avoidance were positively associated with academic burnout (β_1_ = 0.12, *p*_1_ < 0.001; β_2_ = 0.14, *p*_2_ < 0.001), while neither coping style of obedience nor cooperation was associated with academic burnout. The findings indicate that coping styles of competition and avoidance have a partial mediating effect on the relationship between healthy lifestyle and academic burnout.

Based on the aforementioned results, the moderated mediation model proposed in this study was partially supported. The relationship between healthy lifestyle on academic burnout was partially mediated by coping styles of competition and avoidance, which was moderated by gender. Specific values of indirect and direct effects of healthy lifestyle on academic burnout are shown in Table 5. Direct effect of healthy lifestyle for female college students was higher than male college students. The mediating effect of coping style of competition accounted for 14.81% of the total effect in males and 10.81% of the total effect in females. The same pattern was observed for coping style of avoidance, which accounted for 11.11% of the total effect among males and 8.33% of the total effect among females.

**Table 5:** Direct and indirect effects of healthy lifestyle on academic burnout.

Mediator	Gender		Effect value	Boot standard error	95%CI	Relative effect value
Competition	Male	Total effect	−0.27			
		Direct effect	−0.23	0.02	[−0.28, −0.19]	85.19%
		Indirect effect	−0.04	0.01	[−0.05, −0.03]	14.81%
	Female	Total effect	−0.37			
		Direct effect	−0.33	0.02	[−0.37, −0.28]	89.19%
		Indirect effect	−0.04	0.01	[−0.05, −0.03]	10.81%
Avoidance	Male	Total effect	−0.27			
		Direct effect	−0.24	0.02	[−0.29, −0.20]	88.89%
		Indirect effect	−0.03	0.01	[−0.04, −0.02]	11.11%
	Female	Total effect	−0.36			
		Direct effect	−0.33	0.02	[−0.38, −0.29]	91.67%
		Indirect effect	−0.03	0.01	[−0.04, −0.02]	8.33%

## 4 Discussion

The current study explored the psychological mechanisms of college students’ healthy lifestyle and academic burnout using dormitory conflict coping styles and gender on the basis of Conservation of Resources Theory and Transactional Theory of Stress. The results revealed that all variables showed significant gender differences except obedience. Specifically, females’ lifestyle was healthier than those of males, and their academic burnout was lower than those of males. Males used coping styles of competition and avoidance more frequently than females did, while they used coping style of cooperation less frequently than females did. According to the perspective of evolutionary psychology, the competition among males for gene copy makes males more aggressive than females (Wyman et al., 2012). Men are more inclined to adopt competition coping style when encountering interpersonal conflict, whereas they more inclined to adopt avoidance coping style to reduce resource consumption and potential risks when competition fails.

The results further revealed that academic burnout was positively correlated with coping styles of competition and avoidance, and negatively correlated with coping style of cooperation and healthy lifestyle. Importantly, the relationship between healthy lifestyle on academic burnout was moderated by gender, which was partially mediated by coping styles of competition and avoidance. These findings support all the proposed assumptions.

Firstly, the results of the current study revealed that college students’ healthy lifestyle was negatively correlated with academic burnout, supporting Hypothesis 1. The findings are in line with those of previous studies on physical exercise and academic burnout (e.g., Fu et al., 2023; Rehman et al., 2024). In the present study, healthy lifestyle measured not only physical exercise, but also healthy diet, health responsibility, regular life, health hazards, interpersonal behavior, stress management and life appreciation. Thus, the present study provides additional evidence for the relationship between academic burnout and multidimensional healthy lifestyle among general college students.

The findings suggest that the healthier college students’ lifestyle is, the lower their level of academic burnout, supporting the Conservation of Resources Theory (Hobfoll, 1989). According to this theory, healthy lifestyle, such as healthy diet, regular exercise, and harmonious interpersonal relationships, tends to reduce perceived stress. For Chinese students, maintaining regular exercise could reduce academic burnout and depressive symptoms and positively impact students’ mental health (Chen et al., 2022; Ye et al., 2024). Healthy lifestyle therefore is less taxing on individuals’ psychological resources and physical energy and then could effectively alleviate academic burnout. Individuals with unhealthy lifestyles, such as irregular diet, break night, and a lack of exercise, tend to exert large amounts of psychological resources and physical energy, resulting in a state of resource depletion and higher levels of academic burnout. In addition, according to Person-Context Interaction Theories (Beasley et al., 2013), lifestyle, as an environmental factor, has a considerable impact on emotion and perceived stress. Therefore, increased attention should be given to the role of college students’ lifestyle in their mental health.

Secondly, in addition to adopting a correlational view of the role of healthy lifestyle in academic burnout, the present study aimed to explain this correlation, by testing whether dormitory conflict coping styles could account for the association between healthy lifestyle and academic burnout. Our results showed that college students’ coping styles of competition and avoidance for dormitory conflicts have a partial mediating effect on the relationship between healthy lifestyle and academic burnout, supporting Hypothesis 2. These findings suggest that healthy lifestyle could indirectly reduce the level of academic burnout by reducing the use of competition and avoidance coping styles. According to the Transactional Theory of Stress (Lazarus and Folkman, 1987), when an individual’s lifestyle is unhealthy for a long period of time, the individual may face more psychological and academic stress and then develops negative cognition toward dormitory members. Such negative cognition prompted them to adopt the negative coping styles of competition and avoidance to cope with conflicts with dormitory members. Negative coping styles may aggravate conflict. In this case, individuals should use more psychological resources and physical energy to cope with the conflict, which may aggravate academic burnout. Conversely, individuals with a healthy lifestyle may perceive less stress and are less likely to experience conflicts with dormitory members. Even in the face of conflicts with dormitory members, they may be less likely to adopt the negative coping styles of competition and avoidance. In contrast, they may adopt positive coping styles to effectively solve conflicts, which would alleviate conflicts and reduce the consumption of psychological resources, thereby alleviating the level of academic burnout.

The current study also found that healthy lifestyle was negatively correlated with coping styles of competition and avoidance, and positively correlated with coping styles of obedience and cooperation, consistent with the findings of previous studies (e.g., Fan et al., 2023; Liu et al., 2021). These results suggest that college students with unhealthy lifestyle are more inclined to adopt negative coping styles of competition and avoidance when faced with conflicts with dormitory members, whereas college students with healthy lifestyle are more inclined toward coping style of cooperate or obedience to alleviate or resolve dormitory conflicts.

Thirdly, our results revealed that the association between healthy lifestyle and academic burnout was moderated by gender, supporting Hypothesis 3. For both male and female, direct effect of healthy lifestyle on academic burnout is obvious, and there is a stronger direct effect of healthy lifestyle for female college students. Two interpretations might explain our findings. On the one hand, this effect may be related to gender differences in psychological development. Given that psychological maturity occurs earlier among females than among males, women are good at introspection and self-discipline than men are, and therefore have healthier lifestyle such as diet, nutrition, and self-actualization (Hacıhasanoğlu et al., 2011; Tirodimos et al., 2009). The healthier an individual’s lifestyles are, the lower the perceived psychological stress and academic stress are (Ezoe and Morimoto, 1994; Maruyama and Morimoto, 1997; Zhu et al., 2024). Female college students may have sufficient support for healthy behaviors in the face of academic and campus stresses, and may therefore experience a lower level of academic burnout relative to male college students. On the other hand, it may be related to gender differences in physical activity. Regular physical exercise could reduce the perception of stress (Zhai et al., 2021), which in turn buffers the high level of academic burnout caused by an unhealthy lifestyle. However, male college students tend to have irregular physical activity, which perform many physical activities with high frequency, high intensity and short duration, and may therefore experience a higher level of academic burnout relative to female college students.

Finally, the present findings suggest that academic burnout may be prevented by the following interventions. First, mental health educators can provide healthy life education courses and interpersonal relationship counseling to help college students cultivate healthy lifestyles, establish good interpersonal relationships in dormitories, and learn to adopt positive coping styles to effectively resolve dormitory conflicts, thereby alleviating academic burnout. Second, individual differences in lifestyles and interpersonal relationships are apparent, and interpersonal relationships or interactions differ in different dormitories. For example, male college students have higher levels of interpersonal sensitivity than female college students (Liu X. et al., 2024). Group counseling could be conducted in dormitories to help college students develop a healthy academic and personal lifestyle. Third, the moderating effect of gender on the relationship between academic burnout and healthy lifestyle suggests the need to pay more attention to helping male college students improve their lifestyles and reduce the frequency of negative coping styles such as competition and avoidance. This may reduce their level of academic burnout.

Several limitations that should be considered in future studies. Firstly, because this was a cross-sectional study, it was unable to establish causality or directionality of the effects. Future studies should employ longitudinal designs to clarify the effect of healthy lifestyle on academic burnout over time. Secondly, although the current sample of college students included different majors (e.g., computer technology, arts, and bioengineering), the sample was from one university, which may have introduced sampling bias and affected the generalizability of the findings to other regions. Future studies should include college students from different regions and backgrounds to improve the external validity and generalizability of the research. Thirdly, the Cronbach’s *α* values for the avoidance and obedience subscales in the present study were low. Future studies could develop a new questionnaire or revise the original questionnaire. In addition, although this study examined how coping style influenced the association between healthy lifestyle and academic burnout, potential confounders, such as personality traits and pre-existing mental health conditions, could affect the results. Future studies should consider these potential confounders.

## 5 Conclusion

The present study shows that healthy lifestyle is negatively correlated with academic burnout, which is partially mediated by coping styles of competition and avoidance in dormitory conflicts, and moderated by gender. These findings suggest that college students with healthier lifestyle tend to engage in less coping styles of competition and avoidance for dormitory conflicts, leading to a lower level of academic burnout. Compared with male college students, female college students show a stronger direct effect of healthy lifestyle on academic burnout. The present study provides additional evidence for the importance of the interconnected relationships among coping style, academic burnout and multidimensional healthy lifestyle among general college students, which enriches Conservation of Resources Theory and Transactional Theory of Stress. In addition, we should pay attention to male college students with poor dormitory relations, improve their lifestyles and guide them to adopt positive coping styles to resolve dormitory conflicts. This will help reduce their level of academic burnout.

## Data Availability

The raw data supporting the conclusions of this article will be made available by the authors, without undue reservation.
